# Supplementary material for: Revisiting Date and Party Hubs: Novel Approaches to Role Assignment in Protein Interaction Networks
Source: PLoS Comput Biol. 2010 Jun 17;6(6):e1000817. doi: 10.1371/journal.pcbi.1000817 (PMC2887459; doi:10.1371/journal.pcbi.1000817)
Supplement: Figure S1 — Hub deletion effects for AP/MS-only and Y2H-only data sets. (0.07 MB PDF) [file pcbi.1000817.s001.pdf]

Supplementary Figure S1

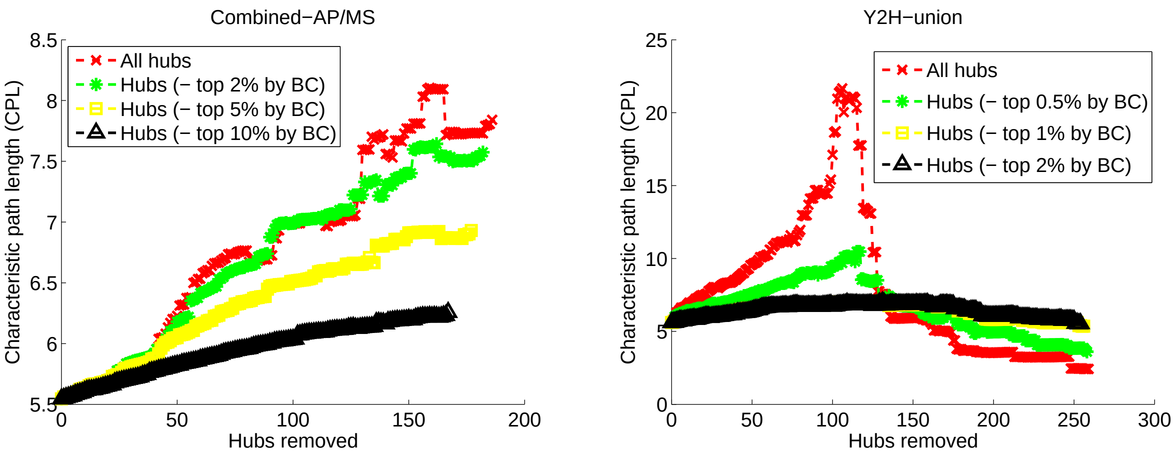

(a) (b)

Figure S1: **Hub deletion effects for AP/MS-only and Y2H-only data sets.** Change in characteristic path length (CPL, the mean length of all finite pairwise shortest paths) on removal of hubs in decreasing order of degree from the (a) 'Combined-AP/MS' and (b) 'Y2H-union' data sets [1]. The 'top X%' captions refer to deletion of all hubs except the X% with the highest betweenness centrality values. Note that when deleting the full sets of hubs, the Y2H network shows a much more dramatic increase in CPL, which may suggest that date hubs are more crucial to network connectivity than party hubs (the Y2H hubs being predominantly date hubs, whereas the AP/MS hubs are mostly party hubs [1]). However, only a very tiny fraction of Y2H-union hubs seem to be responsible for the huge CPL increase on deletion, and protecting these few high-betweenness hubs greatly reduces the impact of hub deletion on network connectivity. This shows that the vast majority of those referred to as date hubs are on average no more central to the network than the party hubs.

References

[1] Yu H, Braun P, Yildirim MA, Lemmens I, Venkatesan K, et al. (2008) High-quality binary protein interaction map of the yeast interactome network. Science 322: 104–110.
