# Supplementary material for: Revisiting Date and Party Hubs: Novel Approaches to Role Assignment in Protein Interaction Networks
Source: PLoS Comput Biol. 2010 Jun 17;6(6):e1000817. doi: 10.1371/journal.pcbi.1000817 (PMC2887459; doi:10.1371/journal.pcbi.1000817)
Supplement: Figure S2 — Topological node role assignments and relation with avPCC. (0.24 MB PDF) [file pcbi.1000817.s002.pdf]

Supplementary Figure S2

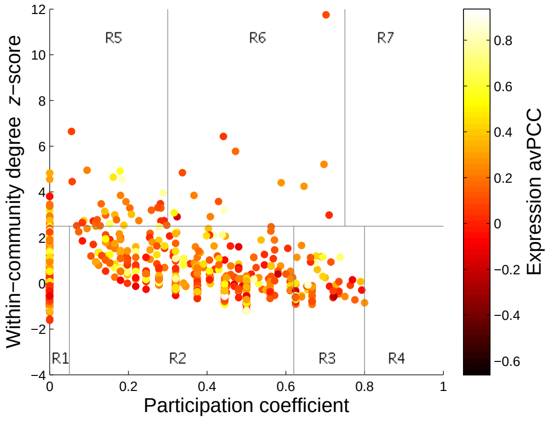

(a) FHC ($\gamma = 0.5$, 35 communities)

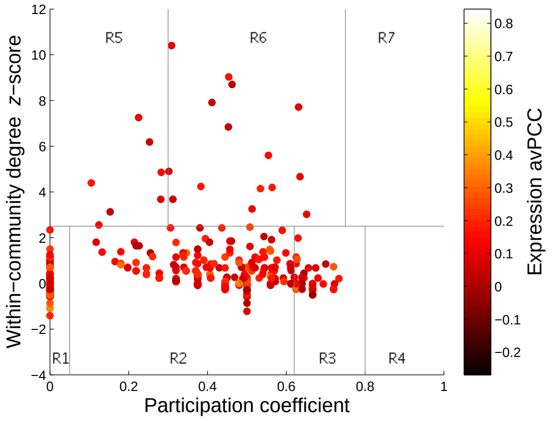

(b) CCSB-HI1 ($\gamma = 0.5$, 32 communities)

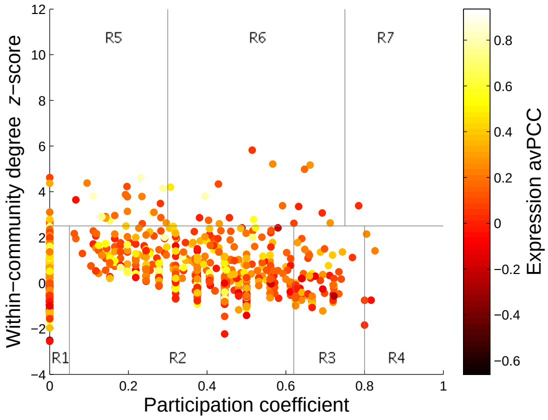

(c) FHC ($\gamma = 2$, 81 communities)

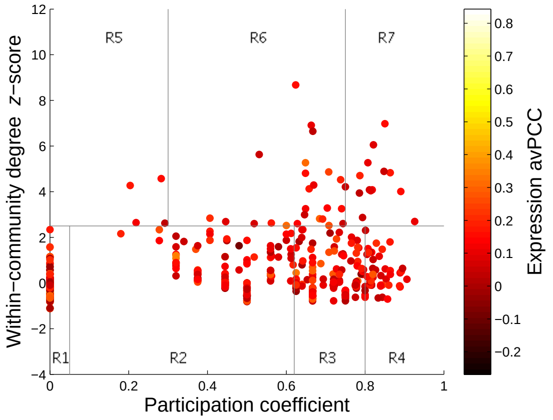

(d) CCSB-HI1 ($\gamma = 2$, 71 communities)

Figure S2: **Topological node role assignments and relation with avPCC.** Plots for (a),(c) Yeast network (FHC [1]—2,233 nodes) and (b),(d) Human network (CCSB-HI1 [2]—1,307 nodes). As in Figure 3, but with different resolution parameter (γ) settings used for community detection (see Text S1).

References

[1] Bertin N, Simonis N, Dupuy D, Cusick ME, Han JDJ, et al. (2007) Confirmation of organized modularity in the yeast interactome. PLoS Biology 5: e153.

[2] Rual JF, Venkatesan K, Hao T, Hirozane-Kishikawa T, Dricot A, et al. (2005) Towards a proteome-scale map of the human proteinprotein interaction network. Nature 437: 1173–78.
