# Supplementary figures and images for: Revisiting Date and Party Hubs: Novel Approaches to Role Assignment in Protein Interaction Networks
Source: PLoS Comput Biol. 2010 Jun 17;6(6):e1000817. doi: 10.1371/journal.pcbi.1000817 (PMC2887459; doi:10.1371/journal.pcbi.1000817)

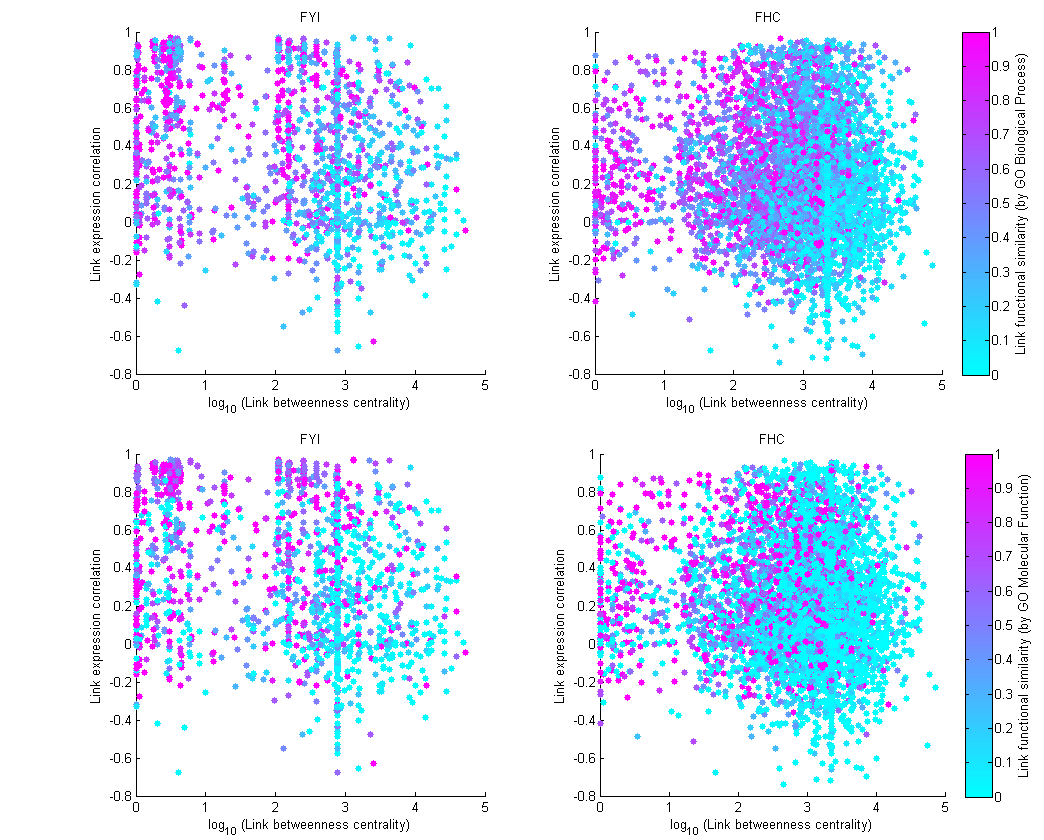

Supplement: Figure S3 — Relating interaction betweenness, co-expression, and functional similarity. Plots show link betweenness centralities versus expression correlations, with points coloured according to average similarity of interactors' GO Biological Process (BP, above) and Molecular Function (MF, below) annotations, for two protein interaction data sets: FYI (778 nodes, 1,798 links) and FHC (2,233 nodes, 5,750 links). Pearson correlation coefficient values of log(link betweenness) with functional similarity are BP: −0.41 (z-score≅−18.6, p-value≅3.9×10^−77^) for FYI, −0.42 (z-score≅−33.9, p-value≅4.7×10−252) for FHC; MF: −0.39 (z-score≅−17.3, p-value≅4.5×10^−67^) for FYI, −0.31 (z-score≅−24.7, p-value≅1.6×10−134) for FHC. (0.15 MB PNG) [file pcbi.1000817.s003.png]
