# Supplementary material for: Revisiting Date and Party Hubs: Novel Approaches to Role Assignment in Protein Interaction Networks
Source: PLoS Comput Biol. 2010 Jun 17;6(6):e1000817. doi: 10.1371/journal.pcbi.1000817 (PMC2887459; doi:10.1371/journal.pcbi.1000817)
Supplement: Figure S4 — Community structure in the largest connected component of the FYI network. (0.07 MB PDF) [file pcbi.1000817.s004.pdf]

Supplementary Figure S4

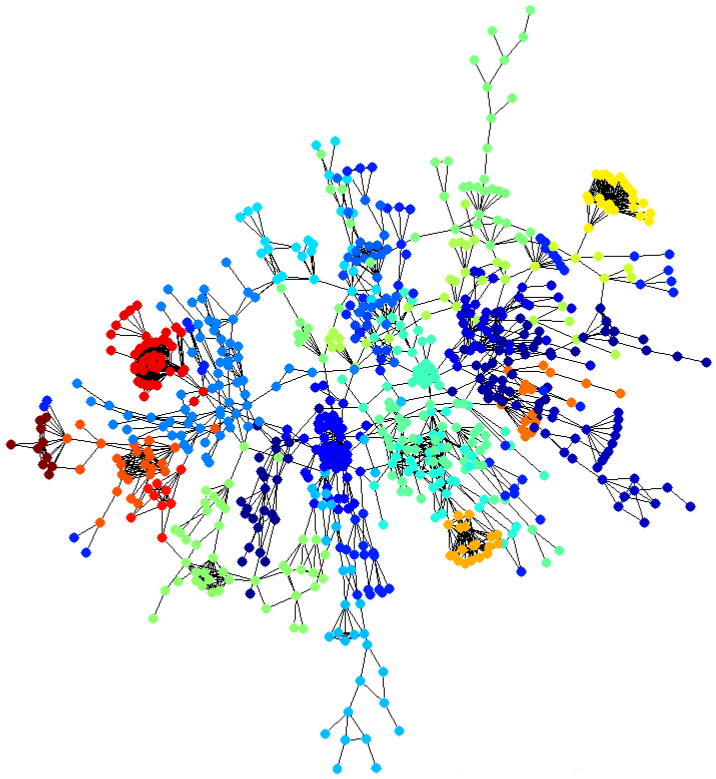

Figure S4: **Community structure in the largest connected component of the FYI network [1].** The different colours correspond to different communities (25 in all). The graph modularity value for this partition is 0.8784. We generated this visualisation using the Kamada-Kawai algorithm [2].

References

[1] Han JDJ, Bertin N, Hao T, Goldberg DS, Berriz GF, et al. (2004) Evidence for dynamically organized modularity in the yeast protein-protein interaction network. Nature 430: 88–93.

[2] Kamada T, Kawai S (1989) An algorithm for drawing general undirected graphs. Information Processing Letters 31: 7–15.
