# Supplementary material for: Revisiting Date and Party Hubs: Novel Approaches to Role Assignment in Protein Interaction Networks
Source: PLoS Comput Biol. 2010 Jun 17;6(6):e1000817. doi: 10.1371/journal.pcbi.1000817 (PMC2887459; doi:10.1371/journal.pcbi.1000817)
Supplement: Table S1 — Evaluating community partitions. (0.02 MB PDF) [file pcbi.1000817.s005.pdf]

Supplementary Table S1

**Data set**	**Commu-nities**	**MF** *IC*			**CC** *IC*			**BP** *IC*			**Best** *IC*		
		Min	Max	Avg	Min	Max	Avg	Min	Max	Avg	Min	Max	Avg
FYI	25	2.05	43.09	14.36	4.28	51.60	17.18	2.99	35.74	15.72	4.81	51.60	20.15
FYI	25 (random)	1.28	2.78	1.88	1.25	3.00	2.07	1.46	3.04	2.13	1.46	3.04	2.36
FHC	63	1.47	51.37	11.22	0.11	68.18	16.40	1.73	98.51	17.08	1.97	98.51	20.08

Table S1: **Evaluating community partitions.** Information Content (IC) of the most enriched term for each of the three GO ontologies (MF – Molecular Function; CC – Cellular Component; BP – Biological Process) and over all three ontologies combined ('Best IC'). We give the minimum, maximum, and average IC over all of the communities (at the default resolution $\gamma = 1$) that we detected in two data sets, FYI [1] and FHC [2]. We generated the random communities for FYI using the same-size distribution to the actual ones. In other words, we remove the actual community labels of all proteins and then randomly re-assign them (using one label per protein).

References

[1] Han JDJ, Bertin N, Hao T, Goldberg DS, Berriz GF, et al. (2004) Evidence for dynamically organized modularity in the yeast protein-protein interaction network. Nature 430: 88–93.

[2] Bertin N, Simonis N, Dupuy D, Cusick ME, Han JDJ, et al. (2007) Confirmation of organized modularity in the yeast interactome. PLoS Biology 5: e153.
