# Supplementary material for: Revisiting Date and Party Hubs: Novel Approaches to Role Assignment in Protein Interaction Networks
Source: PLoS Comput Biol. 2010 Jun 17;6(6):e1000817. doi: 10.1371/journal.pcbi.1000817 (PMC2887459; doi:10.1371/journal.pcbi.1000817)
Supplement: Text S1 — Communities in the Interactome. (0.05 MB PDF) [file pcbi.1000817.s006.pdf]

Supplementary Text S1: Communities in the Interactome

A network consists of elements (called nodes) that are connected to each other by edges (called links). Many real-world networks can be divided naturally into close-knit sub-networks called communities. The investigation of algorithms for detecting communities in networks has received considerable attention in recent years [1, 2].

From an intuitive standpoint, communities should consist of groups of nodes such that there are many links between nodes in the same group but few links between nodes in different groups. To detect communities algorithmically, this notion must be formalised. In order to identify community structure in the various interaction networks that we examine, we employ a method based on optimising the well-known quality function known as graph 'modularity' [3, 4]. Suppose that an unweighted network with n nodes and m links is divided into N communities $C_1, C_2, \cdots, C_N$. Let k_i denote the degree (number of links) of node i and let A be the $n \times n$ adjacency matrix, so that $A(i, j)$ is 1 if nodes i and j have a link between them and 0 if they do not. The modularity Q is then given by [4]

$$Q = \frac{1}{2m} \sum_{l=1}^{N} \sum_{i,j \in C_l} \left(A_{ij} - \frac{k_i k_j}{2m} \right) , \tag{1}$$

where $k_i k_j/(2m)$ is the expected number of links between nodes i and j in a network with the same expected degree distribution but with links placed at random. Graph modularity thus captures how many more links there are within the specified communities than one would expect to see by chance in a network with no modular structure. Note, however, that (1) assumes a particular null model that explicitly preserves the expected degree distribution in the random setting. It is possible to employ other null models [2], though this is the most common choice. In fact, we use an extension of this method based on an analogy to the Potts model in statistical mechanics [5]. This incorporates a resolution parameter (denoted by γ) into the equation for modularity, leading to the quality function

$$H = \frac{1}{2m} \sum_{l=1}^{N} \sum_{i,j \in C_l} \left(A_{ij} - \gamma \frac{k_i k_j}{2m} \right) . \tag{2}$$

Setting $\gamma = 1$ leads to the standard modularity function (1), which is what we use for the results in Figure 3. However, we also present results for $\gamma = 0.5$ and $\gamma = 2$ in Figure S2, demonstrating that whilst the number of communities changes substantially as we increase or decrease the resolution, the pattern of role assignments to

the nodes remains similar.

Using this framework, we can detect communities by maximising the quality function (2) over all possible network partitions. Because this problem is known to be NP-complete [6], reliably finding the global maximum is computationally intractable even for small networks. Thankfully, there exist a number of good computational heuristics that can be used to obtain good local maxima [1, 2, 7]. Here we use recursive spectral bisection [8]. One can use similar procedures to optimise quality functions other than modularity, and the analysis that we have described can be employed in those cases as well.

Maximising graph modularity (1) is expected to give a partition in which the density of links within each community is significantly higher than the density of links between communities. In Figure S4, we show the network partition (with nodes coloured according to community) that results from applying such an optimisation to the largest connected component of the filtered yeast interactome (FYI) data set [9].

To assess how well the obtained topological communities reflect functional organisation, we used annotations from the Gene Ontology (GO) database [10] to define their *Information Content* (IC). GO provides a controlled vocabulary for describing genes and gene products, such as proteins, using a limited set of annotation terms. It consists of three separate ontologies—one each for biological process, cellular component, and molecular function. For each community, we computed the p-value of the most-enriched GO annotation term; the frequency of this term within its community is highest relative to its background frequency in the entire network. To do this, we used the hypergeometric distribution, which corresponds to random sampling without replacement. The extent of enrichment can then be gauged using IC [11], which is defined as

$$IC = -\log_{10}(p)\,, \tag{3}$$

where p denotes the p-value. In Table S1, we summarise the results of calculating this measure for communities detected (for $\gamma = 1$) on two of the yeast interaction data sets, FYI and the more recent filtered high-confidence (FHC [12]). For comparison, we also examine a random partition of FYI into communities with the same size distribution as the actual ones.

It is clear that on average there is very significant functional enrichment within the detected communities. In particular, it is far greater than could be expected by chance. This is in accordance with previous studies on communities in protein interaction networks [13–17]. It is also evident that IC varies widely over communities and that not all of them are equally enriched. There are some relatively heterogeneous communities (which are not aptly described by a single, specific GO term) and others that show a very high functional coherence. In particular, a more detailed inspection of the community composition reveals that proteins that are part of the large and small ribosomal subunit complexes are almost perfectly grouped together, and several other communities consist exclusively of proteins that are known to be part of a given complex. Thus, the topology of

the interaction network provides a great deal of information about the functional organisation of the proteome. We do not claim that our particular partitioning is in any sense unique; rather, it is only a means to an end, as our aim is to examine the implications of community structure for individual protein roles, with particular reference to the notion of date and party hubs. We have also used the greedy algorithm described by Blondel *et al.* [18] as an alternative method for optimising modularity. We obtained results that are very similar to those presented here.

References

[1] Fortunato S (2010) Community detection in graphs. Physics Reports 486: 75–174.

[2] Porter MA, Onnela J-P, Mucha PJ (2009) Communities in networks. Notices of the American Mathematical Society 56: 1082–1097, 1164–1166.

[3] Newman MEJ, Girvan M (2004) Finding and evaluating community structure in networks. Phys Rev E 69: 026113.

[4] Newman MEJ (2006) Modularity and community structure in networks. Proc Natl Acad Sci USA 103: 8577–8582.

[5] Reichardt J, Bornholdt S (2006) Statistical mechanics of community detection. Phys Rev E 74: 016110.

[6] Brandes U, Delling D, Gaertler M, Gorke R, Hoefer M, et al. (2008) On modularity clustering. Knowledge and Data Engineering, IEEE Transactions on 20: 172–188.

[7] Danon L, Diaz-Guilera A, Duch J, Arenas A (2005) Comparing community structure identification. Journal of Statistical Mechanics: Theory and Experiment 2005: P09008.

[8] Newman MEJ (2006) Finding community structure in networks using the eigenvectors of matrices. Phys Rev E 74: 036104.

[9] Han JDJ, Bertin N, Hao T, Goldberg DS, Berriz GF, et al. (2004) Evidence for dynamically organized modularity in the yeast protein-protein interaction network. Nature 430: 88–93.

[10] Ashburner M, Ball CA, Blake JA, Botstein D, Butler H, et al. (2000) Gene ontology: tool for the unification of biology. The Gene Ontology Consortium. Nature Genet 25: 25–29.

[11] Resnik P (1995) Using information content to evaluate semantic similarity in a taxonomy. In: Proc. 14th Int'l Joint Conf. Artificial Intelligence. pp. 448–453.

[12] Bertin N, Simonis N, Dupuy D, Cusick ME, Han JDJ, et al. (2007) Confirmation of organized modularity in the yeast interactome. PLoS Biology 5: e153.

[13] Dunn R, Dudbridge F, Sanderson CM (2005) The use of edge-betweenness clustering to investigate biological function in protein interaction networks. BMC Bioinformatics 6: 39.

[14] Adamcsek B, Palla G, Farkas IJ, Derenyi I, Vicsek T (2006) CFinder: locating cliques and overlapping modules in biological networks. Bioinformatics 22: 1021–1023.

[15] Chen J, Yuan B (2006) Detecting functional modules in the yeast protein-protein interaction network. Bioinformatics 22: 2283–2290.

[16] Maraziotis I, Dimitrakopoulou K, Bezerianos A (2008) An in silico method for detecting overlapping functional modules from composite biological networks. BMC Systems Biology 2: 93.

[17] Lewis ACF, Jones NS, Porter MA, Deane CM (2009) The function of communities in protein interaction networks. E-print arXiv: 0904.0989.

[18] Blondel VD, Guillaume JL, Lambiotte R, Lefebvre E (2008) Fast unfolding of communities in large networks. Journal of Statistical Mechanics: Theory and Experiment 2008: P10008.
